# Surgeons are apprehensive to use DCD lungs despite similar post-transplant outcomes: A 20-year UNOS retrospective analysis

**DOI:** 10.1016/j.jhlto.2024.100185

**Published:** 2024-11-28

**Authors:** J. Sam Meyer, Oliver K. Jawitz, Yury Peysakhovich, Dan Aravot, Matthew G. Hartwig, Yaron D. Barac

**Affiliations:** a The Division of Cardiovascular and Thoracic Surgery, Rabin Medical Center, Petach-Tikva, Israel; b Tel Aviv University, Sackler Faculty of Medicine, Tel Aviv, Israel; c The Division of Thoracic Surgery, Duke University Medical Center, Durham, NC; d Department of Biomedical Engineering, Ben-Gurion University of the Negev, Beer Sheva, Israel

**Keywords:** DCD, DBD, UNOS, Lung transplantation, Organ procurement

## Abstract

**Purpose:**

As rates of lung transplants in the US grow, waitlist mortality increases. While the literature reports similar survival outcomes of DBD and DCD transplants, research should investigate improvements to DCD lung recovery protocols to increase the total number recovered. Recently, Choi et al. presented donor variables indicative of ultimate lung recovery^1^. However, expansion of DCD lung transplants requires a comparison of these indicators to DBD donors for application of similar parameters to increase the rate of DCD lung recovery to ensure that viable DCD organs are not discarded due to overly stringent donor and organ requirements.

**Methods:**

We performed a retrospective analysis of United Network for Organs Sharing (UNOS) Organ Procurement and Transplantation Network/UNOS STAR (Standard Analysis and Research) database. Donors who donated ≥1 organ from 10/1999–01/2019 were extracted and stratified according to DBD and DCD status. Associated characteristics of potential DCD and DBD lung donors were compared, and a multivariable logistic regression model with ≥1 transplanted lung was constructed to evaluate the independent effects of important predictors.

**Results:**

Our data included 179,228 potential lung donors, 162,157 DBD (31,486 donated, 19.4% recovery) and 17,071 DCD (526 donated, 3.1% recovery). Odds of lung non-use between DBD and DCD donors were significantly associated with blood type, alcohol use, cause of death, smoking history, drug use, death circumstance, ethnicity, gender, hypertension, cancer, age, and lung pO2 on 100% P/F ratio (*P* < 0.001 for all variables). A multivariable regression analysis showed that the odds of a potential DCD donating lungs is 75% lower than (*P* < 0.001) that of a potential DBD when the cause of death (COD) is stroke, head trauma (44% lower *P* = 0.076), CNS tumor (22% lower *P* = 0.174) or MVA (69% lower *P* = 0.183). A history of diabetes for over 10 years was strongly associated with non-use for DCD lungs (OR, 0.87, *P* = 0.71), whereas an under 10-year history was associated with increased use (OR 2.33, *P* = 0.008, OR 1.07 *P* = 0.819).

Lungs from donors ages 40–49 are more likely to be procured than those <30 or >50 in both DBD and DCD. However, likelihood of procurement is 1.84 [95% 1.42, 2.38, *p* < 0.001] times higher in 40–49-year-old vs. <30-year-old donors when comparing DBD vs. DCD, and 2.43 [95% 1.83, 3.22, *p* < 0.001] times higher than patients >50 in DBD vs DCD donors. In addition, for each era, the odds for procuring DCD vs. DBD lungs consistently improved [95% 1.46–2.57, *p* < 0.001].

Rejected DCD lungs were associated with donors with higher cardiopulmonary function. Left ventricular ejection fractions in discarded DCD lung donors were higher than those of discarded DBD lung donors (DCD 56.9% ± 13.6 vs. DBD 51.3% ±17.3 *P* = <0.001). Similar non-use patterns were identified for lung PO_2_ on 100% O_2_ (DCD 189.4 ± 121.3 vs. DBD 150.0 ± 106.2 *P* = <0.001), and when the P/F ratio was above 350.00 (DCD 13.5% vs. DBD 7.7% *P* = <0.001).

**Conclusion:**

Despite literature reporting comparable survival of DCD and DBD organs, this study highlights discrepancies in lung procurement practices that evaluate donor characteristics differently in DBD and DCD donors. Further study should investigate whether similar discrepancies exist in the procurement process of other organs.

## Background

Lung transplantation is the gold standard therapy for patients with end-stage lung disease. Patients who undergo single or double lung transplants have an average of 2.8 and 2.4 life-years saved, respectively, compared to patients remaining on the waitlist (median survival 4.2 and 5.4 vs. 2.3 years).^1^ While the rates of lung transplants in the US have grown by 52.3% over the past decade, waitlist mortality still remains high at 14.6 deaths per 100 waitlist years, which yields a one-year survival of 88.8%, and the lowest overall survival amongst all solid organ transplants.^2^ However, this rise in allocation has been outpaced by the increasing demand for transplantation, as advanced lung diseases such as Covid-19, COPD, sarcoidosis, and other fibrotic conditions have led to a record number of new candidates added to the US lung transplantation list in 2019, amidst a decline in transplantation volume due to COVID-19.^2^, ^3^, ^4^

Ongoing scientific and policy efforts have sought to increase the number of deceased lung donors, yielding a 62% increase over the last decade. However, organ availability remains the largest obstacle to reducing waitlist mortality. Across all solid organs, allograft donation after circulatory death (DCD) has increased consistently since 2007, yet the average number of organs transplanted per DBD donor in the US is still nearly double that of DCD donors (3.3 vs. 1.8).^5^ These transplants are mostly limited to livers and kidneys, while lungs are underutilized in the US compared to other western countries.^6^

While historically the first successful organ transplants throughout the 1960s were obtained from a donor after circulatory death, ethical implications surrounding consent for withdrawal of care as well as the logistics of warm ischemic time and other parameters, have made DBD the more common method. However, advances in technology have allowed for the maintenance of sustainable respiration and circulation when a patient’s capacity to breathe spontaneously and maintain circulatory function has been irretrievably lost. These developments, coupled with the increased demand, have led to the non-heart-beating donor (NHBD) Maastricht classification for standardizing protocols to include these potential donors. Updated in Paris at the 6th International Conference on Organ Donation after Circulatory Death in 2013, the definition of controlled DCD refers to planned withdrawal of life support in operating rooms or intensive care units (category III), while uncontrolled DCD is when attempts at donor resuscitation are unsuccessful, or they suffer an unforeseen cardiopulmonary arrest (categories I, II, IV).^7^

In the US today, controlled DCD is the primary mechanism of DCD procurement in order to reduce warm ischemic times, and multicenter-large-cohort studies continue to show comparable survival outcomes between these DCD organs and DBD organs.^8^, ^9^, ^10^, ^11^, ^12^ Yet, despite these favorable outcomes, it is possible that various institutional and ethical barriers, as well as unfamiliarity of surgeons with DCD practices, have led to their underutilization. In this study, we looked to analyze patterns in clinical practice that differentiate non-use in DBD versus DCD lungs to identify potential subjectivity and ultimately promote utilization.

## Patients and methods

### Data source

We performed a retrospective cohort analysis using the United Network for Organs Sharing (UNOS) Organ Procurement and Transplantation Network/UNOS STAR (Standard Analysis and Research) database. The Organ Procurement and Transplantation Network is administered by UNOS under contract with the US Department of Health and Human Services. This database contains data on all transplant candidates undergoing listing for solid organ transplantation in the United States since October 1987. The study was deemed exempt by our Institutional Review Board, IRB N.172 Pro00073879; Informed consent was waived.

### Study design

All donors who donated at least one organ and were admitted between October 1999 and January 2019 were extracted from the UNOS registry and stratified according to DBD and DCD status. Our data consisted of 207,744 patients above the age of 18. Of these patients, 35,015 were excluded because they were missing over 20% of the variables used in our regression model, while 10,572 patients were excluded because they did not consent to lung donation.

### Statistical analysis

Associated characteristics of potential lung donors were compared between DCD and DBD lungs using the Mann-Whitney Wilcoxon test for continuous variables and the Pearson Chi-Square test for categorical variables. A multivariate logistic regression model was constructed with an outcome of at least one lung transplanted, and the independent significant effects of six candidate variables were selected using the Backward Elimination Method. Selected variables included donor age, cause of death (etiologically), history of smoking, physiological mechanism of death, history of diabetes, and gender. Then, based on this variable selection, the interaction of each of the selected variables with the type of death (DBD vs. DCD) was tested in a second block using the Forward Linear Regression Method.

## Results

### Donor characteristics

**Figure 1:**
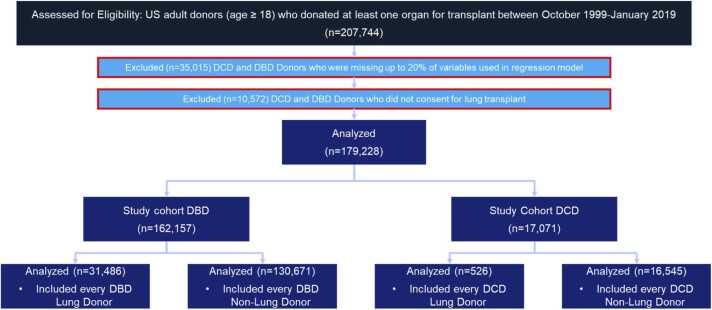
CONSORT diagram of study design. All donors who donated at least 1 organ for transplantation between 1999 and 2019 were identified in the UNOS registry and stratified based on DCD and DBD status. Individual organ-level and donor-level analyses were performed. DCD donors who did not consent for LTx were excluded from further analysis because their lungs could not be evaluated or used for transplantation. Donors missing over 20% of the data were excluded in order to conduct multivariable analysis. DBD, Donation after brain death; DCD, donation after circulatory death.

179,228 potential lung donors met our inclusion criteria. The DBD cohort included 162,157 potential donors, while the DCD cohort included 17,071 potential donors. In the DBD cohort, lung non-use was seen in 80.6% (*n* = 130,671) of these potential donors, while in the DCD cohort, non-use was significantly greater at a rate of 96.9% (*n* = 526). Consequently, the DBD donation rate was significantly higher than the DCD cohort, as 19.4% (n=31,486) of potential DBD lungs were recovered while just 3.1% (*n* = 526) of DCD lungs were recovered (Figure 1).

A higher representation of patients who had a history of alcohol abuse (DCD 22.1% vs DBD 17.5%), a history of cancer (DCD 92.5% vs DBD 91.1% *P* = <0.001), a history of smoking (DCD 78.2% vs. DBD 62.9% *P* = <0.001), and a history of illicit drug use (DCD 65.5% vs DBD 44.7% *P* = <0.001) were seen in non-used DCD lungs when compared to non-used DBD lungs. Surprisingly, DCD lungs that were rejected for transplantation were associated with donors with higher cardiopulmonary function. For example, left ventricular ejection fractions in discarded DCD lung donors were higher than those of discarded DBD lung donors (DCD 56.9% ± 13.6 vs. DBD 51.3% ±17.3 *P* = <0.001). Similar non-use patterns were identified for lung PO_2_ on 100% O_2_ (DCD 189.4 ± 121.3 vs. DBD 150.0 ± 106.2 *P* = <0.001), and when the P/F ratio was above 350.00 (DCD 13.5% vs. DBD 7.7% *P* = <0.001) (Table 1).

**Table 1:** Donor Characteristics Stratified by DBD and DCD and Use for Transplantation

	Transplanted					Non-Transplanted				
Donor Variables	DBD (n=31,486)		DCD (n=526)		*P Value*	DBD (n=130,671)		DCD (n=16,545)		*P Value*
Donor Age (Yrs)	32.7	(14.6)	36.3	(13.4)	0.001	40.7	(19.3)	39.1	(15.8)	<0.001
Donor Gender					0.396					<0.001
Female	12648.0	(40.2%)	202.0	(38.4%)		51293.0	(41.4%)	5456.0	(34.8%)	
Male	18788.0	(59.8%)	324.0	(61.6%)		72543.0	(58.6%)	10239.0	(65.2%)	
BMI	25.2	(5.3)	26.4	(5.7)	0.007	26.8	(7.0)	27.9	(7.3)	<0.001
Blood Type					0.280					<0.001
A	11382.0	(36.2%)	194.0	(37%)		46167.0	(37%)	6126.0	(39%)	
AB	698.0	(2.2%)	4.0	(1%)		4554.0	(4%)	554.0	(4%)	
B	3381.0	(10.8%)	46.0	(8.7%)		14625.0	(11.8%)	1730.0	(11.0%)	
O	15975.0	(50.8%)	282.0	(53.6%)		58488.0	(47.2%)	7282.0	(46.4%)	
Alcohol Abuse	3150.0	(13.8%)	109.0	(21.3%)	<0.001	13417.0	(17.5%)	3143.0	(22.1%)	<0.001
History Of Cancer	170.0	(0.5%)	2.0	(0.4%)	0.477	1039.0	(91.1%)	98.0	(92.5%)	<0.001
History Of Smoking	3789.0	(53.9%)	33.0	(91.7%)	<0.001	32964.0	(62.9%)	3268.0	(78.2%)	<0.001
History Of Cocaine	1647.0	(56.1%)	33.0	(46.5%)	0.006	6936.0	(54.1%)	993.0	(43.6%)	<0.001
History Of Other Drug Use	6815.0	(55.9%)	118.0	(68.2%)	0.001	21534.0	(44.7%)	3694.0	(65.5%)	<0.001
Donor Ethnicity					<0.001					<0.001
White	20266.0	(64.5%)	428.0	(81.4%)		86073.0	(69.5%)	13026.0	(83.0%)	
Black	5542.0	(17.6%)	39.0	(7.4%)		17590.0	(14.2%)	1133.0	(7.2%)	
Hispanic	4398.0	(14.0%)	42.0	(8.0%)		15624.0	(12.6%)	1167.0	(7.4%)	
Asian	797.0	(2.5%)	11.0	(2.1%)		2617.0	(2.1%)	233.0	(1.5%)	
Other	433.0	(1.4%)	6.0	(1%)		1932.0	(2%)	136.0	(1%)	
History Of Diabetes	1790.0	(5.7%)	32.0	(6%)	0.009	12836.0	(10%)	1307.0	(8%)	<0.001
History Of Hypertension	6189.0	(19.8%)	112.0	(21.5%)	0.348	41358.0	(33.8%)	4418.0	(28.3%)	<0.001
Left Ventricular Ejection Fraction (%)	52.1	(14.4)	58.1	(12.0)	<0.001	51.3	(17.3)	56.9	(13.6)	<0.001
Lung Po2/FiO2	358.0	(150.4)	387.8	(144.5)	0.020	150.0	(106.2)	189.4	(121.3)	<0.001
Lung Po2 On 100%	358.0	(150.4)	387.8	(144.5)	<0.001	150.0	(106.2)	189.4	(121.3)	<0.001
Duration Of Resuscitation (Min)	18.6	(16.6)	14.6	(8.0)	0.0 12	22.3	(18.4)	24.0	(20.6)	0.206
Lung Po2 On 100%) P/F Ratio (Binned)					0.008					<0.001
< 250.00	141.0	(27.0%)	5875.0	(21.7%)		11989.0	(85.8%)	57384.0	(75.1%)	
250.00 - 299.99	13.0	(2.5%)	666.0	(2.5%)		468.0	(3.4%)	4755.0	(6.2%)	
300.00 - 349.99	39.0	(7.5%)	1629.0	(6.0%)		432.0	(3.1%)	3942.0	(5.2%)	
350.00+	329.0	(63.0%)	18915.0	(69.8%)		1081.0	(7.7%)	10324.0	(13.5%)	
Donor Age (Binned)					<0.001					<0.001
<30	15332.0	(48.8%)	184.0	(35.0%)		37812.0	(30.5%)	4712.0	(30.0%)	
30−39	5492.0	(17.5%)	112.0	(21.3%)		16720.0	(13.5%)	2447.0	(15.6%)	
40−49	5497.0	(17.5%)	127.0	(24.1%)		23194.0	(18.7%)	3555.0	(22.7%)	
<=50	5115.0	(16.3%)	103.0	(19.6%)		46109.0	(37.2%)	4981.0	(31.7%)	
Era					<0.001					<0.001
Oct1999-Dec2004	8855.0	(28.2%)	12.0	(2.3%)		49239.0	(39.8%)	1522.0	(9.7%)	
Jan2005-Dec2009	6769.0	(21.5%)	77.0	(14.6%)		27709.0	(22.4%)	3446.0	(22.0%)	
Jan2010-Dec2014	8480.0	(27.0%)	128.0	(24.3%)		26137.0	(21.1%)	5188.0	(33.1%)	
<=Jan2015	7332.0	(23.3%)	309.0	(58.7%)		20751.0	(16.8%)	5539.0	(35.3%)	

In addition, significant differences in donor characteristics between transplanted DBD and DCD cohorts were observed for the following factors: donor age was significantly younger in the transplanted DBD group (32.7 years vs 36.3 years, *P* = 0.001). The representation of male donors was higher in the transplanted DCD cohort (61.6% vs 59.8%, *P* < 0.001), and BMI was significantly higher in the transplanted DCD group (26.4 vs 25.2, *P* = 0.007). The blood type distribution differed significantly between the groups, with a higher proportion of blood type O donors in the transplanted DBD cohort (50.8% vs 53.6%, *P* < 0.001). Ethnicity also showed significant differences, with a higher proportion of White donors in the transplanted DCD group (81.4% vs 69.5%, *P* < 0.001) (Table 1).

For non-transplanted donors, significant differences were observed in the following characteristics: donor age was significantly older in the non-transplanted DCD group (39.1 years vs 40.7 years, *P* < 0.001). A higher proportion of male donors was found in the non-transplanted DCD cohort (65.2% vs 58.6%, *P* < 0.001). The ethnicity of donors showed a higher proportion of White donors in the non-transplanted DCD group (83.0% vs 69.5%, *P* < 0.001) (Table 1).

#### DCD vs. DBD causes and mechanisms of death

Lung non-use was also strongly associated with various donor causes and mechanisms of death, however, these associations differed between the DBD and DCD cohorts. The largest proportion of *non-transplanted* DCD lungs came from donors who died of anoxia likely from withdrawal of support (43.4%) compared with 22.3% of DBD (*P*=<0.001), Similarly, non-use lungs from donors who died of suicide account for 10.9% of DCD compared to 7.2% in DBD non-use (*P*=<0.001). While lungs from donors who had died of natural causes made up 34.5% of non-use in DCD compared to 31.7% in DBD (*P*=<0.001). Complete donor causes and mechanisms of death are presented in Table 2.

**Table 2:** Donor Causes and Mechanisms of Death Stratified by DBD and DCD and Use for Transplantation

	Transplanted					Non-Transplanted				
Donor Variables	DBD (n=31,486)		DCD (n=526)		*P- Value*	DBD (n=130,671)		DCD (n=16,545)		*P-Value*
Donor Cause of Death					<0.001					<0.001
Anoxia	4804.0	(15.3%)	195.0	(37.1%)		27611.0	(22.3%)	6804.0	(43.4%)	
Cerebrovascular/Stroke	10322.0	(32.8%)	131.0	(24.9%)		50027.0	(40.4%)	3200.0	(20.4%)	
Head Trauma	15342.0	(48.8%)	187.0	(35.6%)		42737.0	(34.5%)	4795.0	(30.6%)	
CNS Tumor	263.0	(0.8%)	1.0	(0.2%)		811.0	(0.7%)	44.0	(0.3%)	
Other	704.0	(2.2%)	12.0	(2.3%)		2640.0	(2.1%)	852.0	(5.4%)	
Circumstance Of Death					<0.001					<0.001
Death From Natural Causes	8143.0	(25.9%)	175.0	(33.3%)		38452.0	(31.1%)	5421.0	(34.5%)	
MVA	6680.0	(21.3%)	80.0	(15.2%)		22514.0	(18.2%)	3045.0	(19.4%)	
Suicide	4496.0	(14.3%)	109.0	(20.7%)		8927.0	(7.2%)	1716.0	(10.9%)	
Homicide	3233.0	(10.3%)	26.0	(4.9%)		5845.0	(4.7%)	458.0	(2.9%)	
Child-Abuse	108.0	(0.3%)	0.0	(0.0%)		1642.0	(1.3%)	51.0	(0.3%)	
Accident, Non-MVA	3071.0	(9.8%)	81.0	(15.4%)		12500.0	(10.1%)	1870.0	(11.9%)	
None Of The Above	5704.0	(18.1%)	55.0	(10.5%)		33948.0	(27.4%)	3134.0	(20.0%)	
Mechanism Of Death					<0.001					<0.001
Death From Natural Causes	403.0	(1.3%)	6.0	(1.1%)		1909.0	(1.5%)	632.0	(4.0%)	
Drowning	96.0	(0.3%)	1.0	(0.2%)		1287.0	(1.0%)	246.0	(1.6%)	
Seizure	314.0	(1.0%)	7.0	(1.3%)		930.0	(0.8%)	186.0	(1.2%)	
Drug Intoxication	1581.0	(5.0%)	47.0	(8.9%)		5929.0	(4.8%)	1096.0	(7.0%)	
Asphyxiation	977.0	(3.1%)	60.0	(11.4%)		3934.0	(3.2%)	1204.0	(7.7%)	
Cardiovascular	1702.0	(5.4%)	68.0	(12.9%)		13892.0	(11.2%)	3636.0	(23.2%)	
Electrical	11.0	(0.0%)	0.0	(0.0%)		63.0	(0.1%)	21.0	0.1%)	
Gunshot Wound	6117.0	(19.5%)	60.0	(11.4%)		9994.0	(8.1%)	974.0	(6.2%)	
Stab	64.0	(0.2%)	1.0	(0.2%)		199.0	(0.2%)	34.0	(0.2%)	
Blunt Injury	8613.0	(27.4%)	119.0	(22.6%)		29865.0	(24.1%)	3745.0	(23.9%)	
SIDS	6.0	(0.0%)	0.0	(0.0%)		183.0	(0.1%)	12.0	(0.1%)	
Intracranial Hemorrhage/Stroke	10778.0	(34.3%)	147.0	(27.9%)		52005.0	(42.0%)	3296.0	(21.0%)	
Gunshot/Stab Wound	6.0	(0.0%)	0.0	(0.0%)		20.0	(0.0%)	4.0	(0.0%)	
None of the above	767.0	(2.4%)	10.0	(1.9%)		3618.0	2.9%)	609.0	(3.9%)	

#### Reasons for organ discard

**Figure 2:**
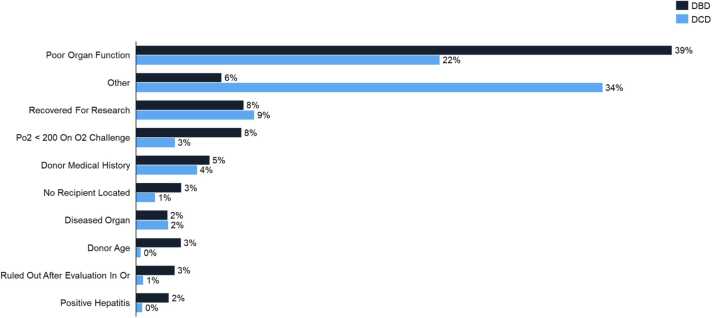
Comparing the leading reasons for organ discard between DBD (n=130,671) and DCD (n=16,545) donors. Transplant teams are required to indicate in the OPTN assessment of organs for transplantation the specific reasons for organ discard.

When evaluating organs for transplant, transplantation teams are required to complete an OPTN assessment form, and, if relevant, to select from 38 possible reasons for discard. Of these reasons, “poor organ function” was the most cited reason for discarding DBD donor lungs (39.1%). However, when evaluating potential DCD lungs, transplantation teams most frequently selected the option “other” (34.1%). A comparison of the most indicated reasons for discard is represented in Figure 2, and a full list of these indicators is shown in Table 3.

**Table 3:** Reasons for Organ Discard (Non-Use) Stratified by DBD and DCD

Non-Transplanted				
Reason for Organ Discard	DBD (n=130.670)		DCD (n=16,546)	
Poor Organ Function	51131	39.1%	3670	22.2%
Recovered For Research	10281	7.9%	1431	8.6%
Po2 < 200 On O2 Challenge	10069	7.7%	472	2.9%
Other	8181	6.3%	5637	34.1%
Donor Medical History	7038	5.4%	739	4.5%
No Recipient Located	4341	3.3%	234	1.4%
Donor Age	4291	3.3%	61	0.4%
Ruled Out After Evaluation in OR	3706	2.8%	91	0.5%
Diseased Organ	3020	2.3%	390	2.4%
Positive Hepatitis	3147	2.4%	80	0.5%
Organ Refused by All National Program	3107	2.4%	229	1.4%
Infection	2172	1.7%	114	0.7%
Trauma To Organ	1890	1.4%	125	0.8%
Time Constraints	1069	0.8%	877	5.3%
Donor Quality	1781	1.4%	141	0.9%
Organ Refused by All Regional Program	1607	1.2%	138	0.8%
Donor Social History	1521	1.2%	128	0.8%
Hemodynamically Unstable Donor	1313	1.0%	282	1.7%
Medical Examiner Restricted Recovery	940	0.7%	103	0.6%
Cardiac Arrest	192	0.1%	498	3.0%
Positive Gram Stain	665	0.5%	23	0.1%
History Of Lung Disease (Valid For Lu)	617	0.5%	64	0.4%
Recovered For Transplant Was Submit For Research	612	0.5%	49	0.3%
Recovered For Transplant Was Discarded Locally	465	0.4%	96	0.6%
Anatomical Abnormalities (Not Valid For Pa Or Pa Segments)	269	0.2%	7	0.0%
Vascular Damage	84	0.1%	1	0.0%
Positive Htlv – 1	76	0.1%	4	0.0%
Biopsy Findings	73	0.1%	1	0.0%
No Local Recovery Team	62	0.0%	9	0.1%
Positive Hiv	27	0.0%	1	0.0%
Surgical Damage In Or	21	0.0%	0	0.0%
Ejection Fraction < 50%	20	0.0%	0	0.0%
Ruled Out Due To Biopsy	15	0.0%	0	0.0%
Exported, Not Transplanted Or Transplant Unknown	10	0.0%	1	0.0%
Donor Abo	7	0.0%	1	0.0%
Past Medical History	26	0.0%	1	0.0%
Emotional	1	0.0%	0	0.0%
Missing Data	6823	5.2%	848	5.1%

#### Multivariable regression analysis

**Figure 3:**
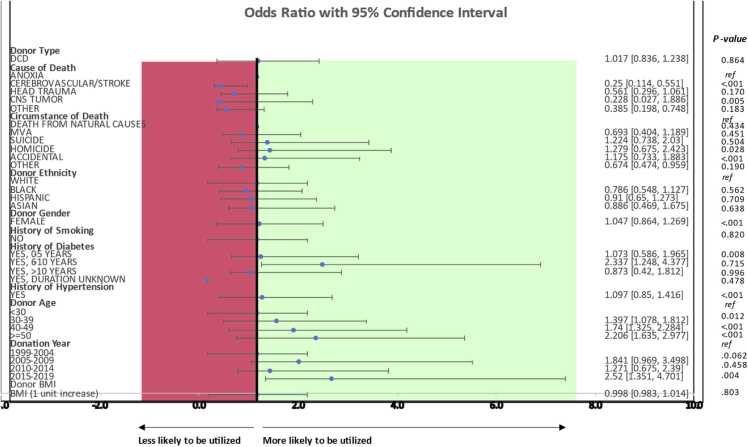
Forest plot of donor factors leading to lung non-use for DBD and DCD donors. A multivariable analysis was conducted to identify the independent effect of DBD and DCD death on various donor characteristics that lead to non-use.

A multivariable regression analysis was used to evaluate the unique impact of DCD on determining the likelihood of organ transplantation, using an interaction term between the type of death and the cause of death. The overall interaction effect was found to be significant (*p*=0.003). The odds of a potential DCD donor donating lungs is 75% less than (*P*<0.001) that of a potential DBD donor when the cause of death (COD) is stroke, 44% less (*P*=0.076) when COD is head trauma, and 22% less (*P*=0.174) when COD stems from a CNS tumor. Similarly, DCD donors who die in motor vehicle accidents are 69% (*P*=0.183) less likely to have their organs procured when compared to DBD donors. A history of diabetes for over 10 years was strongly associated with non-use for DCD lungs (OR, 0.87, *P*=0.71), whereas an under 10-year history was associated with increased use (OR 2.33, *P*=0.008, OR 1.07 *P*=0.819). Similarly, the most recent era, 2015–2019, was associated with an increase in use (OR, 2.52; *P*=0.003). These odds ratios are represented in the forest plot in Figure 3.

## Discussion

In this retrospective analysis of the UNOS database from 1999 to 2019, we analyzed DCD and DBD donor factors associated with the non-use of donated lungs. We demonstrated significant differences in odds of procurement between these two cohorts when it comes to donor characteristics, causes and mechanisms of death, and reasons for discard indicated by clinical teams.

To our knowledge, this is the first study to compare pre-donation and donation characteristics of DBD and DCD lungs that were transplanted using a large national transplant database.

Comparing the general trends of lung transplantation, Van Raemdonck et al. and OPTN data described an upward trend in DCD lung procurement from only three in 2003 to 190 in 2020. This *6233% increase* over the course of just 17 years, compared to the *119% increase* in DBD lung procurement over the same period (from 1085 in 2003 to 2371 in 2020), highlights the success of policy changes, technological breakthroughs, and surgical advances that have enabled this rapid growth of DCD organ procurement.^10^, ^13^, ^14^ However, the large discrepancy between total DCD vs. DBD lung donors also underscores the tremendous unmet potential and ongoing organ waste occurring in this field. Moreover, even with this rapid growth in DCD transplantation, the recovery rates of DBD have improved from 15.2% to 26.1% between 1999 and 2019, while DCD has only moved from 0.8%−5.3, indicating a need to improve efficiency in this process.

Overall, among DCD donors in the US, there was only a 3.1% lung recovery rate from donors who donated another organ, compared to 19.4% for DBD donors. This underutilization is not only low when compared to US DBD lungs, but when compared to other DCD lungs in other western countries as well. Lomero *et al.* evaluated DCD organ recovery among 35 countries in the European Committee on Organ Transplantation of the Council of Europe and found that between 2008–2016, every country that had a lung DCD protocol had significantly greater recovery rates. These include Belgium (50.2%), Netherlands (38.2%), Switzerland (29.6%), Ireland (14.3%), Austria (11.6%), the United Kingdom (10.9%), and Spain (9.4%). ^6^

Our data revealed that transplantation teams applied more rigorous criteria when evaluating many donor characteristics for procurement of DCD lungs compared to DBD lungs and recorded these decisions in the OPTN. These discrepancies in what transplant teams deemed acceptable were evident in both the lifestyle and clinical characteristics of these donors, including history of alcohol abuse, smoking, and illicit drug use, as well as history of cancer and Pao_2_/Fio_2_ ratios above 350.00 (Table 1). Donor Pao_2_/Fio_2_ ratios of under 350 have been correlated with recipient death within one year of lung transplantation; however, there is no evidence to suggest an increased risk of death in DCD over DBD lungs.^15^ Notably, another donor characteristic strongly associated with recipient complications is age, as donors above the age of 60 have lower 5- and 10-year survival rates; however, our data does not indicate any major differences in non-use in this category.^16^

The ongoing challenge of organ shortage in the US is continuously improving. Since 2005, the lung allocation score (LAS) has shortened median wait times and decreased waitlist deaths.^17^ Ex vivo lung perfusion (EVLP) has given surgeons critical time to assess lungs with uncertain quality and even provide therapeutics that can increase their function, all while they continue to be ventilated and perfused with a protein and electrolyte solution.^18^ Other interventions such as bioengineered lungs that use recipient cells grown on decellularized lung scaffolds,^19^ as well as xenotransplantation from various pigs and primates, although still allusive in the literature, may provide more scalable solutions to current organ shortages in general and lungs in particular.^20^

Despite the growing scientific evidence, the transplant community has been slow to incorporate DCD donors into the standard of care for numerous reasons. Logistically, DCD procurements require increased levels of cooperation between various surgical teams and organ procurement authorities, and issues of familial consent play a bigger role. Donor management has also proven to be an obstacle to DCD procurement, as the inevitable occurrence of warm ischemic time has been shown to increase the risk of graft failure and primary graft dysfunction.^21^ However, as our study shows, it is possible that the greatest obstacle towards increasing the donor pool with utilizing DCD lungs is the institutional hesitancy of transplant teams to utilize these viable organs simply because they lack experience in doing so.

Hints of this reluctance to use DCD lungs can be found in our data. When a surgeon assesses the viability of an organ and ultimately decides to discard it, the OPTN form requires teams to explain their reasoning. Of the nearly 40 options provided on the form, the most common reason for discard in DBD lungs is poor organ function, a relatively objective measure which accounts for the lung anatomy, physiology, and predicted ability to ultimately perform in a recipient’s body. However, when it comes to DCD lungs, a general sense of discomfort may be leading to more subjective decisions to discard. Whereas in the case of DBD lungs, surgeons deem the organ unfeasible due to poor organ function 39.1% of the time, in DCD lungs, they select this reason only 22.2% of times. Rather, the most common reason marked by surgeons for discard of DCD lungs is “other” (34.1%) (Figure 2). This subjective reasoning may indicate that surgeons are not specifically wary of underlying anatomical conditions, donor history, infections, or other clinical indicators listed on the form, but instead are uncomfortable due to a general sense of inexperience and unfamiliarity.

Recently, Siddique et al. similarly outlined many of the aforementioned challenges in DCD lung procurement and recommended increased data transparency, education amongst surgeons, consensus in national policies, and optimization of donor management to improve utilization.^22^ We believe that studies such as this one, as well as increased patient-outcome studies from centers around the world, can lead to increased recognition of DCD donor lungs as a significant, underused, and safe source of donor lungs.

## Study Limitations

There are inherent limitations in conducting a long-term retrospective analysis of the UNOS database due to missing data. As the UNOS network was founded in 1984 and data collection methods continued to improve through the years, many data points we now deem critical to both donor and recipient monitoring do not exist in the database. Furthermore, retrospective analyses, in general, cannot account for unmeasured confounders that cannot be considered. The database itself is also not able to give the total picture of potential DBD and DCD donors, considering records are only kept on patients who have donated at least one organ. The characteristics of donors who did not donate at all, a cohort much larger than our sample, are not represented here. Despite this, the UNOS/OPTN database represents the largest database for transplantation in the world and is largely considered to be the gold standard for understanding organ procurement practices both in the US and globally.

## Conclusion

Our research shows that while DCD organ procurement is on the rise in the US, the percentage of procured lungs from potential donors is significantly lower in DCD patients compared to DBD patients despite similar post-transplant survival. The ambiguous reason for DCD lung discard demonstrates that DCD lung transplantation is still not the standard of care, likely related due to surgeons’ apprehensiveness and discomfort in transplanting these lungs. Hopefully, as trends of DCD lung use continue to rise, the recovery rate will grow in turn as surgeons grow accustomed to these procurement practices. Studies like these and exercises to build consensus can help accelerate these efforts.

## Ethical Approval

The study was deemed exempt by our Institutional Review Board, IRB N.172 Pro00073879 approved on 5/29/16; Informed consent was waived.

## Consent

All authors consented to publication of this manuscript. Consent to participate was not required according to the design of this study.

## Authors Contribution

JSM, and YDB designed the study and reviewed the literature. JSM, conducted statistical analysis and analyzed the data. JSM, MGH, and YDB were the main authors. All authors critically read and approved the final manuscript.

## Financial Disclosure

N/A.

## Availability of Data and Materials

Data are available upon request from the corresponding author.

## Declaration of Competing Interest

The authors declare that they have no known competing financial interests or personal relationships that could have appeared to influence the work reported in this paper.
